# Journal of Ovarian Research reviewer acknowledgement 2013

**DOI:** 10.1186/1757-2215-7-13

**Published:** 2014-04-09

**Authors:** Sham S Kakar, Stefano Palomba, Benjamin K Tsang, David T Curiel

**Affiliations:** 1 Department of Physiology and Biophysics, University of Louisville, Louisville KY 40202, USA; 2 Department of Obstetrics, Gynecology and Pediatrics, Sterility Centre P. Bertocchi, Obstetrics and Gynecology Unit, A.O. S.Maria Nuova, IRCCS, Reggio Emilia and University of Modena, Reggio Emilia, Italy; 3 Department of Obstetrics & Gynaecology, University of Ottawa and the Chronic Disease Program, Ottawa Hospital Research Institute, Ottawa K1H 8L6, Canada; 4 Cancer Biology Division, School of Medicine, Washington University in St. Louis, St. Louis MO 63108, USA

## Abstract

**Contributing reviewers:**

The editors of Journal of Ovarian Research would like to thank all of our reviewers who have contributed to the journal in volume 6 (2013).

## 

Ronit Abir

Israel

Pedro Acién

Spain

Raffaele Addeo

Italy

Laurie Ailles

Canada

Ayman Al-Hendy

United States of America

Adolfo Allegra

Italy

Carlo Alviggi

Italy

Stavroula Baka

Greece

Shisan Bao

Australia

Sharmila Bapat

India

Animesh Barua

United States of America

Susan Bellis

United States of America

Sinan Berkman

Turkey

Deepa Bhartiya

India

Mariusz Bidzinski

Poland

Zachariah Bobby

India

Fernando Bonilla-Musoles

Spain

Chinmoy Bose

India

Andrew Bradford

United States of America

Pedro Caballero-Campo

United States of America

Valentina Cascini

Italy

Neeraj Chawla

India

Ri-Cheng Chian

Canada

Nam Hoon Cho

South Korea

Fabrice Cognasse

France

Denise Connolly

United States of America

Shantanu Deshpande

India

Laura Diaz Cueto

Mexico

Jacques Donnez

Belgium

Toshiaki Endo

Japan

Jeong Min Eom

South Korea

Angela Falbo

Italy

David Feder

Brazil

Lingda Feng

China

Gabriella Ferrandina

Italy

Christina Fotopoulou

United Kingdom

John Fruehauf

United States of America

Norbert Gleicher

United States of America

Victor Gomel

Canada

Angelique Goverde

Netherlands

Jedrzejewski Grzegorz

Poland

Ilknur Gumus

Turkey

Tayfun Gungor

Turkey

Hua Guo

United States of America

Digant Gupta

United States of America

Micah Hill

United States of America

Roy Homburg

Israel

HeFeng Huang

China

Kaisa Huhtinen

Finland

Krishna Jagarlamudi

United States of America

Venkatakrishna Jala

United States of America

Kannamannadiar Jayaprakasan

United Kingdom

Ze-Xu Jiao

United States of America

Joshua Johnson

United States of America

Zheng Junch

China

Evanthia Kandarakis

Greece

Selvendiran Karuppaiyah

United States of America

Hidetaka Katabuchi

Japan

Nadeem Khan

United States of America

Tae Jin Kim

South Korea

Yun Hwan Kim

South Korea

Junpei Kimura

Japan

Endre Kiss-Toth

United Kingdom

Carolyn Klinge

United States of America

Gulengul Köken

Turkey

Ikuo Konishi

Japan

Pamela Kreeger

United States of America

Lydia Kriegl

Aruba

Kuang-Tai Kuo

Taiwan

Mertihan Kurdoglu

Turkey

Hiroaki Kurihara

Japan

Robert J. Kurman

United States of America

Antonio La Marca

Italy

Giovanni Battista La Sala

Italy

Joseph Lau

United States of America

David Lee

United States of America

Jung Hun Lee

South Korea

Marie-Claude Leveille

Canada

Chang-Zhong Li

China

Elaine Lin

United States of America

Gen-Min Lin

Taiwan

Shanling Liu

China

Felicity Lose

Australia

Emilio Lucia

Italy

Fernando Luiz Affonso Fonseca

Brazil

Mohammad Malik

United States of America

Vincenzo Dario Mandato

Italy

Santos Mañes

Spain

Udo R. Markert

Germany

Maurie Markman

United States of America

John Martignetti

United States of America

Axel Methner

Germany

Takashi Minegishi

Japan

Bruce Murphy

Canada

Daniela Murtas

Italy

Toyofumi Naka

Japan

Kenneth Nephew

United States of America

Ceana Nezhat

United States of America

Paul Nguewa

Spain

Gabriela M Oprea-Ilies

United States of America

Francesco Orio

Italy

Makoto Orisaka

Japan

Raoul Orvieto

Israel

Shoji Oura

Japan

Nuri Ozturk

United States of America

Giuseppina Padova

Italy

Lynn Parker

United States of America

Divya Patel

United States of America

Sushmita Pathy

India

Gilberto Paz Filho

Australia

Tanja Pejovic

United States of America

Chun Peng

Canada

Terhi Piltonen

Finland

Vito Pistoia

Italy

Ricardo Pommer

Chile

Renaud Prudent

France

Tilman Rachner

Germany

Mariusz Ratajczak

United States of America

Mojtaba Rezazadeh Valojerdi

Iran

Tyvin A Rich

American Samoa

Rodney Rocconi

United States of America

Peter Rose

United States of America

Rosaria Ruggeri

Italy

Nobuyuki Sakurai

Japan

Berna Seckin

Turkey

Ellora Sen

India

Kok-Min Seow

Taiwan

Francesco Sesti

Italy

John Henry Shepherd

United Kingdom

Yuan-Wei Shih

Taiwan

Masayuki Shimada

Japan

Yimin Shu

United States of America

Arti Shukla

United States of America

Elvio Silva

United States of America

Ajay Singh

United States of America

Rajesh Singh

United States of America

Shailesh Singh

United States of America

Johan Smitz

Belgium

Janusz Solski

Poland

Heidi Sowter

United Kingdom

Clare Stevinson

United Kingdom

Colin Stewart

Australia

Norihiro Sugino

Japan

Ying Sun

United Kingdom

Suchinda Taepongsorat

Thailand

Pierosandro Tagliaferri

Italy

Russell Taichman

United States of America

Shailendra Tamaskar

India

Hiroshi Tamura

Japan

Dun-Xian Tan

United States of America

Premal Thaker

United States of America

Howard H Ting

Australia

Fabian Trillsch

Germany

Toshio Tsubota

Japan

Anne Van Langendonckt

Belgium

Jeffery Vancem

United Kingdom

Barbara Vanderhyden

Canada

De Leo Vincenzo

Italy

Irma Virant-Klun

Slovenia

Radu Vladareanu

Romania

Yinsheng Wan

United States of America

He Wang

China

Hongmei Wang

China

Liwei Wang

China

Weihua Wang

United States of America

Gerburg Wulf

United States of America

Lei Xi

United States of America

Xuefeng Xia

United States of America

Ali Yavuzcan

Turkey

Hui Yu

China

Alain Zeimet

Austria

Pumin Zhang

United States of America

Ye Zhang

United States of America

Chengquan Zhao

United States of America

Lingjun Zhao

United States of America

Min Zhao

China

Zhi-Qing Zhao

United States of America

Xiaodong Zhou

United States of America

Shien Zhu

China

Stephanie C. Ziehr

Austria

Anca Zimmermann

Germany

Surekha Zingde

India

Fulvio Zullo

Italy

